# PET-CT has low specificity for mediastinal staging of non-small-cell lung cancer in an endemic area for tuberculosis: a diagnostic test study (LACOG 0114)

**DOI:** 10.1186/s12885-018-5233-5

**Published:** 2019-01-03

**Authors:** Gustavo Werutsky, Bruno Hochhegger, José Antônio Lopes de Figueiredo Pinto, Jeovany Martínez-Mesa, Mara Lise Zanini, Eduardo Herz Berdichevski, Eduardo Vilas, Vinícius Duval da Silva, Maria Teresa Ruiz Tsukazan, Arthur Vieira, Leandro Genehr Fritscher, Louise Hartmann, Marcos Alba, Guilherme Sartori, Cristina Matushita, Vanessa Bortolotto, Rayssa Ruszkowski do Amaral, Luís Carlos Anflor Junior, Facundo Zaffaroni, Carlos H. Barrios, Márcio Debiasi, Carlos Cezar Frietscher

**Affiliations:** 1 Latin American Cooperative Oncology Group (LACOG), Ipiranga Avenue 6681, 99A, Room 806, Porto Alegre, Brazil; 2 Medical School, Pontifical Catholic University of Rio Grande do Sul, Porto Alegre, Brazil; 3 IMED, School of Medicine, Passo Fundo, Brazil; 4 Brain Institute of Rio Grande do Sul, Porto Alegre, Brazil; 5 Hospital São Lucas, Pontifical Catholic University of Rio Grande do Sul, Porto Alegre, Brazil

**Keywords:** Non-small cell lung cancer, PET-CT, Mediastinal staging, Granulomatous infectious diseases

## Abstract

**Background:**

The present study aims to assess the performance of 18F-FDG PET-CT on mediastinal staging of non-small cell lung cancer (NSCLC) in a location with endemic granulomatous infectious disease.

**Methods:**

Diagnostic test study including patients aged 18 years or older with operable stage I-III NSCLC and indication for a mediastinal lymph node biopsy. All patients underwent a 18F-FDG PET-scan before invasive mediastinal staging, either through mediastinoscopy or thoracotomy, which was considered the gold-standard. Surgeons and pathologists were blinded for scan results. Primary endpoint was to evaluate sensitivity, specificity and positive and negative predictive values of PET-CT with images acquired in the 1st hour of the exam protocol, using predefined cutoffs of maximal SUV, on per-patient basis.

**Results:**

Overall, 85 patients with operable NSCLC underwent PET-CT scan followed by invasive mediastinal staging. Mean age was 65 years, 49 patients were male and 68 were white. One patient presented with active tuberculosis and none had HIV infection. Using any SUV_max > 0 as qualitative criteria for positivity, sensitivity and specificity were 0.87 and 0.45, respectively. Nevertheless, even when the highest SUV cut-off was used (SUV_max ≥5), specificity remained low (0.79), with an estimated positive predictive value of 54%.

**Conclusions:**

Our findings are in line with the most recent publications and guidelines, which recommend that PET-CT must not be solely used as a tool to mediastinal staging, even in a region with high burden of tuberculosis.

**Trial registration:**

The LACOG 0114 study was registered at ClinicalTrials.gov, before study initiation, under identifier NCT02664792.

## Background

Lung cancer is the leading cause of cancer-related death in the world. It is responsible for 1,350,000 new cases and 1,180,000 deaths annually worldwide [1]. In Brazil, the incidence of lung cancer is also rising, accounting for approximately 28,000 new cases and 24,500 deaths yearly, according to the most recent report from INCA, the Brazilian National Institute of Cancer [2].

Despite recent advances in terms of early diagnosis achieved with low-dose Computed Tomography (CT) screening, most cases of lung cancer are still diagnosed at late clinical stages (CS), IIIb or IV. In Brazil, approximately 70% of patients present with locally advanced or metastatic disease [3]. Accurate staging of patients with non-small-cell lung cancer (NSCLC) is critical for defining the best treatment modality and predicting prognosis [4]. In the absence of distant metastasis, the status of mediastinal lymph nodes plays a critical role for treatment decisions. In this clinical scenario, the identification of positive mediastinal nodes changes the treatment option from surgery to multimodality treatment approach [5].

Since 2003, PET scan (Positron Emission Tomography) with 18F-fluorodeoxyglucose (18F-FDG) is recommended for NSCLC staging due to its high sensitivity to detect cancer [6, 7]. Currently, PET-CT is the gold-standard procedure for the non-invasive staging of NSCLC patients because it has the capability of identifying distant metastasis that would pass unnoticed in CT, preventing over 30% of unnecessary thoracotomies [8].

Invasive staging of the mediastinal nodes with mediastinoscopy is still the standard of care and the value of PET-CT for this indication is debatable. Previous studies showed sensitivity ranging from 77 to 90% and specificity of 86% for PET-CT in detecting the spread of NSCLC to the mediastinal lymph nodes [9]. One of the most important problems with the use of PET-CT in this situation are the false positives findings. It occurs because 18F-FDG is not a tumor specific agent and other conditions such as granulomatous diseases might present with a high 18F-FDG uptake [10–12].

The aim of this study is to validate PET-CT performance on mediastinal staging of patients with NSCLC living in an endemic area of tuberculosis.

## Methods

### Trial design

The present study is a diagnostic test study designed to evaluate PET-CT performance on the diagnosis of metastatic mediastinal lymph nodes compared with the gold-standard invasive staging with biopsy in patients with non-small cell lung carcinoma.

### Patients

Patients were recruited from the department of Thoracic Surgery at Hospital São Lucas, a tertiary hospital supported by the Public Health System in southern Brazil. Patients aged 18 years or older were eligible if they had newly diagnosed or highly suspected NSCLC and indication for mediastinal lymph node biopsy based on current practice guidelines for staging. All patients were considered to have operable stage I-III disease after initial evaluation (medical history, physical examination and contrast-enhanced CT scan of the chest and upper abdomen). Exclusion criteria were any prior treatment for NSCLC (surgery, chemotherapy or radiotherapy), confirmed distant metastases, pregnancy (women in childbearing age had to agree to taking contraceptive measures and present a negative pregnancy test), altered hematologic and biochemical function.

### Procedures

Patients included in the study were first subjected to a PET-CT scan, which were obtained using integrated PET–CT system (GE Discovery 600) as follows: after a 6-h fast, 18F-FDG was given intravenously with activity of 10 to 15 mCi. Images were acquired 1 and 2 h after the administration of 18F-FDG. Patients were scanned from the head to the upper thigh. A diagnostic CT scan, obtained with the use of a standard protocol (80 to 100 mA, 120 kV, a tube-rotation time of 0.5 s per rotation, a pitch of 6, and a slice thickness of 5 mm, with 70 ml of intravenous contrast medium containing 300 mg of iodine per milliliter [Ultravist, Bayer Schering], administered at a rate of 2.5 ml per second), preceded the PET scan (a 5-min emission scan per table position and 25 min total). The PET scan was reconstructed by filtered back-projection and ordered-subset expectation-maximization (OS-EM), with data from the CT scan used for attenuation correction. Results were evaluated by a radiologist and a nuclear medicine specialist. The maximum standardized uptake value (SUV_max) of the primary tumor was measured and calculated by the software according to standard formulas. Mediastinal lymph node stations were considered positive for metastatic spread if they exhibited focally increased FDG uptake higher than the normal background activity (activity > background), as determined by qualitative analysis. After the PET-CT scan, invasive mediastinal staging was performed. Mediastinoscopy or thoracotomy were considered valid invasive mediastinal staging procedures and were chosen according to surgeon’s discretion. The surgical team was blinded to PET-CT’s results.

### Statistical analysis

The finding of mediastinal lymph nodes with increased 18F-FDG uptake on PET/CT was compared with pathological examination of lymph nodes obtained in the invasive staging procedure. The study primary endpoint was to evaluate PET-CT’s performance with images acquired in the 1st hour of the exam protocol in a per-patient basis. The secondary endpoints were determining PET-CT performance in the 2nd hour of the exam protocol, and evaluate PET-CT’s performance in the 1st hour of the exam protocol evaluated per-nodal station basis (2R, 2 L, 4R, 4 L and 7). Categorical variables were described as their count and percentage. Numerical variables were described as their median, minimum and maximum. Sensitivities, specificities and predictive values were calculated using predefined cutoffs of maximal SUV. The 95% confidence intervals were calculated for sensitivity, specificity and predictive values. A receiver-operating-characteristic (ROC) analysis was performed on the PET-CT per-patient results. In order to obtain a minimal sensitivity and specificity of 90%, while expecting a 40% rate of positive lymph nodes, we estimated that 89 patients would be needed accepting a two-sided type I error of 5%. Assuming a 10% ineligibility rate, the total sample size was 100 patients. Statistical analysis was performed with the use of SPSS software, version 18, and SAS software, version 9.4.

## Results

### Baseline characteristics

**Fig. 1:**
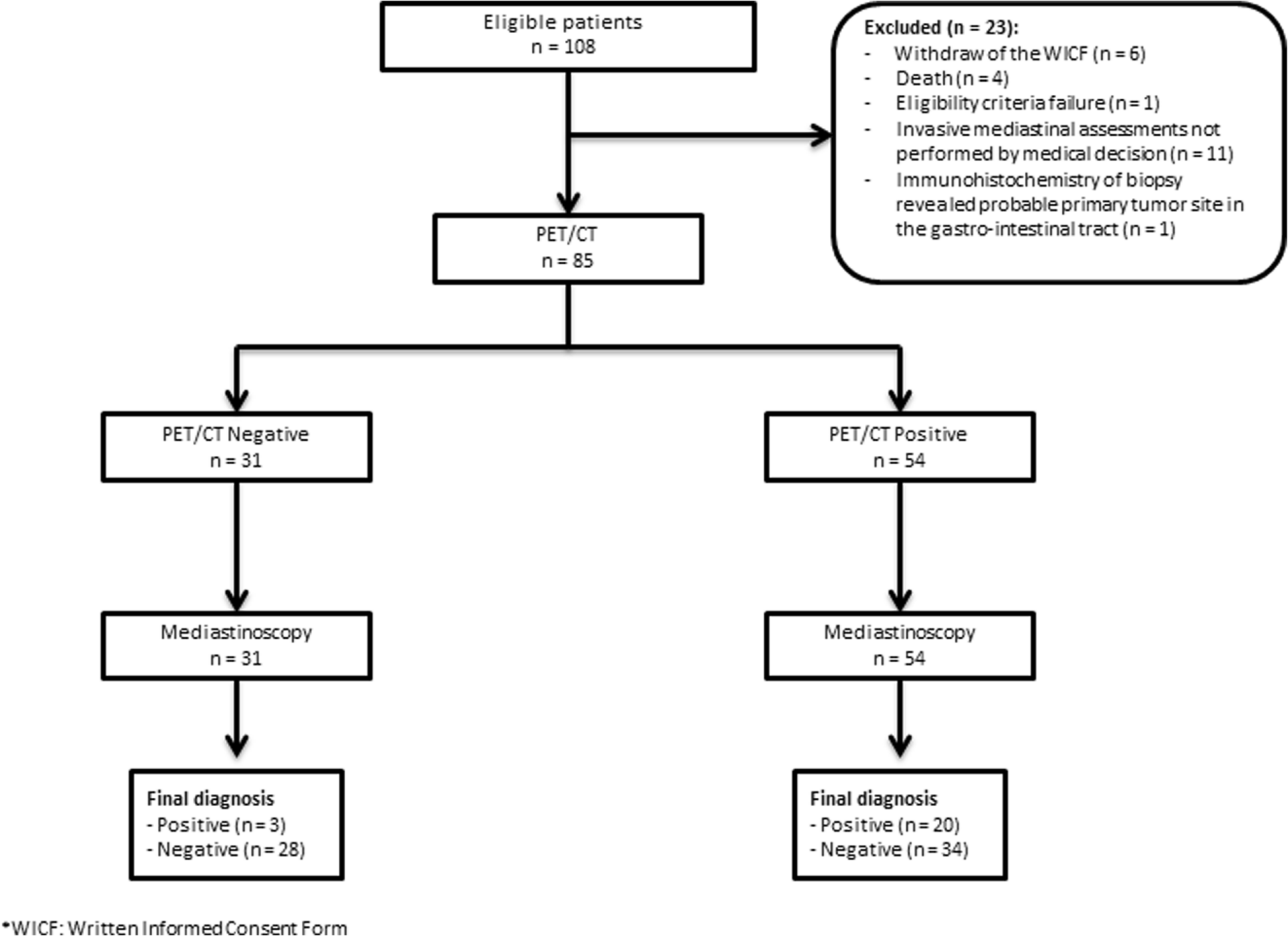
STARD flow diagram for the evaluation of 18F-FDG PET-CT on mediastinal staging of non-small cell lung cancer

From August 2014 to August 2016, 108 patients were enrolled in the study. Eight patients did not perform the PET-CT scan. Of the remaining 100 patients, 85 underwent mediastinal sampling biopsy (by mediastinoscopy or surgery) after PET­CT and they were considered for primary analysis. The STARD flow diagram is shown in Fig. 1.

Table 1 shows baseline characteristics of eligible patients who performed mediastinal sampling. Median age was 65 years, 57.6% patients were male and 80.0% were white. Current or former smokers accounted for 94.1% of the sample, with a high tobacco exposure (median of 45 pack-years). Although the relatively high incidence of tuberculosis in Brazil, only one patient had known TB infection and no patient had an HIV infection.

**Table 1:** Patient’s characteristics at baseline

Characteristic	N (%) or median (min-max) (*n* = 85)
Age (years)	65.0 (47.0–80.0)
Sex	
Male	49 (57.6%)
Female	36 (42.4%)
Race	
White	68 (80.0%)
Black	7 (8.2%)
Other	10 (11.8%)
Smoking status	
Current	35 (41.2%)
Former	45 (52.9%)
Never	5 (5.9%)
Tobacco Exposure (Pack-year)^a^	45.0 (8.1–120.0)
Comorbities	
Hypertension	40 (47.1%)
Diabetes	11 (12.9%)
COPD	30 (35.3%)
Asthma	9 (10.6%)
Active Tuberculosis	1 (1.2%)
HIV positive	0 (0.0%)

Data is presented here as mean (minimum-maximum) or absolute (relative) frequencies. ^a^This analysis takes into account only the 79 patients that were smokers or former smokers

### Comparison of PET-CT and mediastinal invasive staging

From 85 patients, only 23 patients (27.1%) had pathological mediastinal involvement. Of these, PET-CT correctly identified 20 patients (86.9%) that showed an increased uptake of 18F-FDG. Conversely, PET-CT showed increased uptake in the mediastinum of 34 patients that were later on not confirmed to have pathological mediastinal involvement (false-positive rate of 54.8%). 28 out of 62 patients who did not have lymph node involvement on histological analysis did not have increased uptake on PET CT (true-negative rate of 45.2%). When a higher cut-off was used (SUV max ≥5), the false-positive rate reduced to 21.0% and the true-negative rate increased to 79.0%. For the same SUV cut-offs, we found an increased FDG uptake in hour 2, although this difference may not be clinically relevant. When evaluated the 212 available nodal stations, 38 of them had pathological mediastinal involvement (17.9%). Of those 38, PET-CT correctly identified 25 of them (65.8%). 128 out of 174 lymph nodes who did not have involvement on histological analysis did not have increased uptake on PET CT (true-negative rate of 73.6%). Considering SUV max ≥5, the true-positive rate decreased to 47.4% and the true-negative rate increased to 91.4% (Tables 2 and 3).

**Table 2:** PET-CT findings and pathological evaluation of mediastinal lymph nodes after surgical staging (per-patient and per-nodal-station)

PET-CT SUV cut-off	Pathological evaluation of mediastinal lymph nodes	PER-PATIENT (n = 85)				PER-NODAL-STATION (*n* = 212)	
		PET-CT				PET-CT	
		HOUR 1		HOUR 2		HOUR 1	
		Positive	Negative	Positive	Negative	Positive	Negative
SUV_Max^a^ > 0	**Positive**	20	3	20	3	25	13
	**Negative**	34	28	35	27	46	128
SUV_Max^a^ ≥ 2.5	**Positive**	18	5	19	4	23	15
	**Negative**	30	32	30	32	40	134
SUV_Max^a^ ≥ 3	**Positive**	16	7	18	5	21	17
	**Negative**	24	38	26	36	32	142
SUV_Max^a^ ≥ 5	**Positive**	15	8	16	7	18	20
	**Negative**	13	49	18	44	15	159
SUV_Max^a^ ≥ SUV Liver	**Positive**	18	5	18	4	–	–
	**Negative**	23	37	25	34	–	–

^a^SUV_Max: Maximum value of SUV uptake between 2R, 2 L, 4R, 4 L, 7 and aortopulmonary when evaluating per-patient, and 2R, 2 L, 2R, 4 L and 7 when evaluating per-nodal-station

**Table 3:** Sensitivity and specificity of PET-CT using different maximum SUV cutoffs for the staging of the mediastinal lymph nodes (per-patient and per-nodal-station)

Cut-off	Measure	Hour 1 (Per-Patient)	Hour 2 (Per-Patient)	Hour 1 (Per-Nodal-Station)
SUV_Max^a^ > 0	Sensitivity	0.87 (0.66–0.97)	0.87 (0.66–0.97)	0.66 (0.49–0.80)
	Specificity	0.45 (0.33–0.58)	0.44 (0.31–0.57)	0.74 (0.66–0.80)
	Positive Predictive Value	0.37 (0.31–0.44)	0.36 (0.30–0.43)	0.35 (0.28–0.43)
	Negative Predictive Value	0.90 (0.76–0.97)	0.90 (0.75–0.96)	0.91 (0.86–0.94)
SUV_Max^a^ ≥ 2.5	Sensitivity	0.78 (0.56–0.93)	0.83 (0.61–0.95)	0.61 (0.43–0.76)
	Specificity	0.52 (0.39–0.65)	0.52 (0.39–0.65)	0.77 (0.70–0.83)
	Positive Predictive Value	0.38 (0.30–0.46)	0.39 (0.32–0.47)	0.37 (0.28–0.46)
	Negative Predictive Value	0.87 (0.74–0.94)	0.89 (0.76–0.95)	0.90 (0.86–0.93)
SUV_Max^a^ ≥ 3	Sensitivity	0.70 (0.47–0.87)	0.78 (0.56–0.93)	0.55 (0.38–0.71)
	Specificity	0.61 (0.48–0.73)	0.58 (0.45–0.71)	0.82 (0.75–0.87)
	Positive Predictive Value	0.40 (0.31–0.50)	0.41 (0.33–0.50)	0.40 (0.30–0.50)
	Negative Predictive Value	0.84 (0.74–0.91)	0.88 (0.76–0.94)	0.89 (0.85–0.92)
SUV_Max^a^ ≥ 5	Sensitivity	0.65 (0.43–0.84)	0.70 (0.47–0.87)	0.47 (0.31–0.64)
	Specificity	0.79 (0.67–0.88)	0.71 (0.58–0.82)	0.91 (0.86–0.95)
	Positive Predictive Value	0.54 (0.40–0.67)	0.47 (0.36–0.59)	0.55 (0.40–0.68)
	Negative Predictive Value	0.86 (0.78–0.92)	0.86 (0.77–0.92)	0.89 (0.85–0.92)
SUV_Max^a^ ≥ SUV Liver	Sensitivity	0.78 (0.56–0.93)	0.82 (0.60–0.95)	–
	Specificity	0.62 (0.48–0.74)	0.58 (0.44–0.70)	–
	Positive Predictive Value	0.44 (0.35–0.54)	0.42 (0.34–0.51)	–
	Negative Predictive Value	0.88 (0.77–0.94)	0.90 (0.77–0.96)	–

^a^SUV_Max: Maximum value of SUV uptake between 2R, 2 L, 4R, 4 L, 7 and aortopulmonary when evaluating per-patient, and 2R, 2 L, 2R, 4 L and 7 when evaluating per-nodal-station

As showed in Table 3, the highest sensitivity (87%) was observed for the SUV_max > 0 cut-off. The negative predictive value (NPV) using this cut-off was 90%, which wasn’t changed for the images acquired in hour 2. By contrast, in the scenario with the highest specificity, when only uptake with SUV ≥ 5 was considered positive, we found a positive predictive value of only 54%. When using the liver FDG uptake as cut-off for SUV positivity, the sensitivity and specificity was not improved

**Fig. 2:**
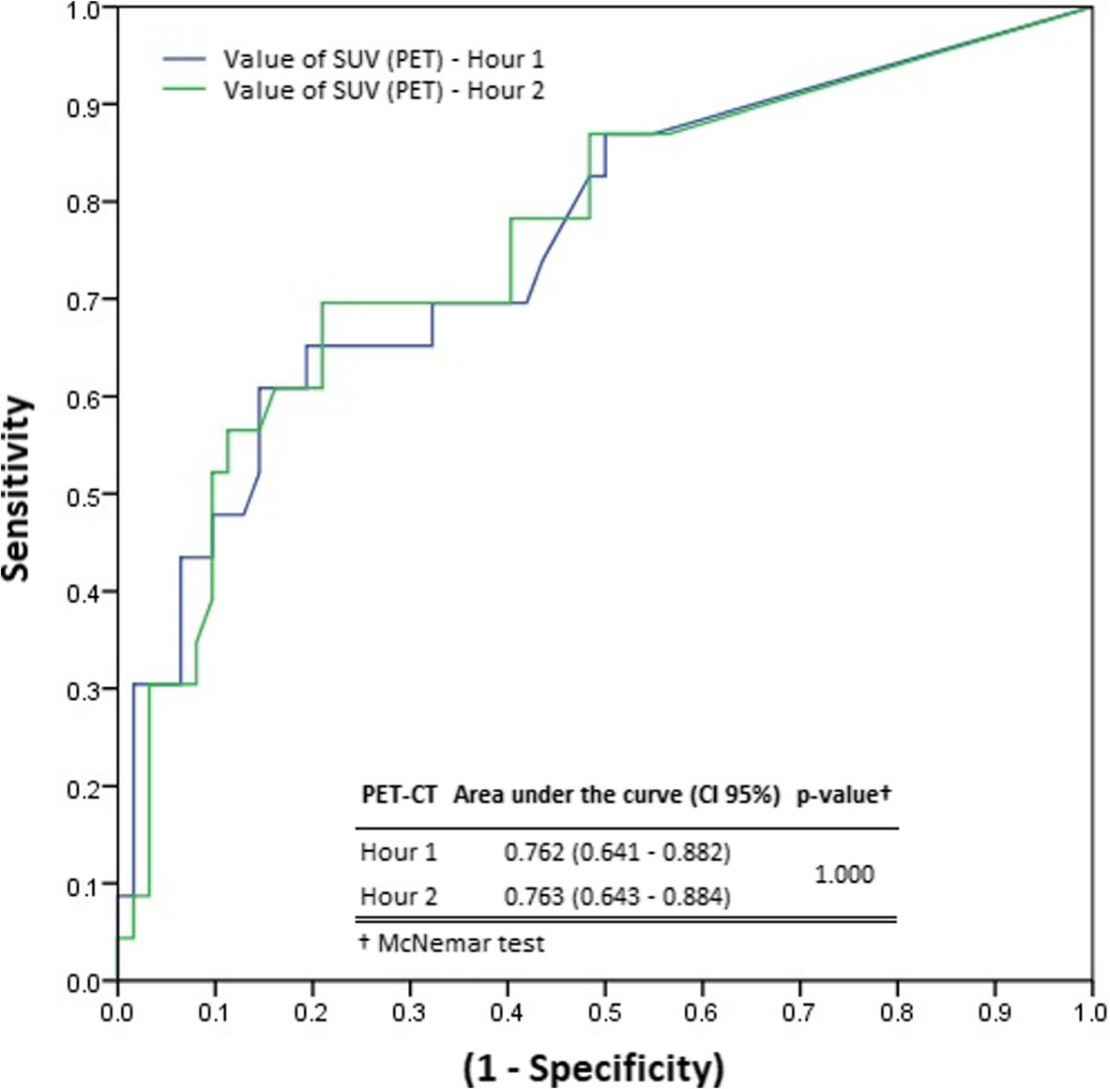
ROC curve comparing PET-CT performance for images acquired in hour 1 and 2

The image acquisition in hour 2 of the protocol did not change the accuracy of the test using ROC (Receiver Operator Characteristic) curve (Fig. 2).

**Fig. 3:**
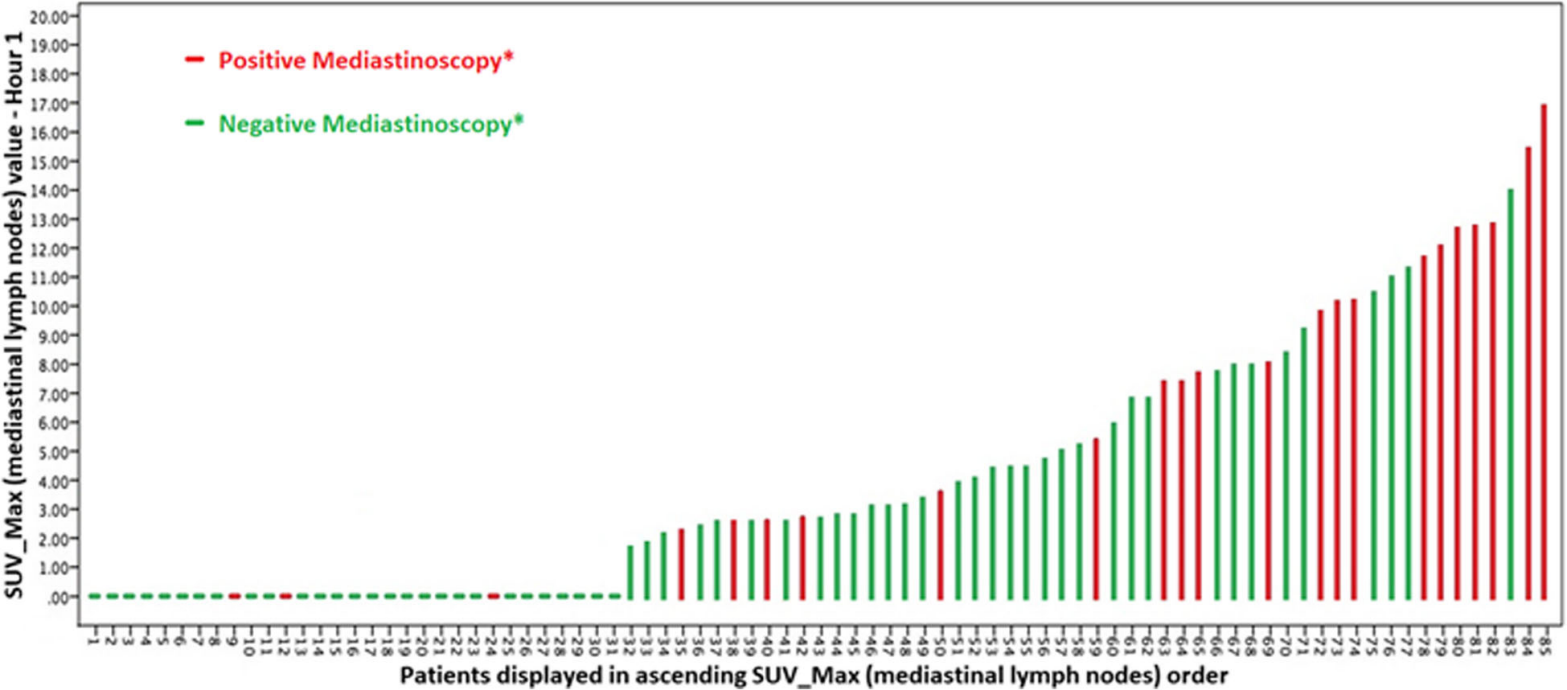
Correlation between maximum SUV and anatomopathological finding for the mediastinal lymphnodes

Among 31 patients with no uptake in mediastinum (SUV = 0), 3 (9.6%) had metastatic lymph node involvement after mediastinal invasive staging (Fig. 3).

## Discussion

Therapeutic options for patients with non-metastatic potentially resectable NSCLC are mainly determined by the presence or absence of mediastinal lymph node metastases (N2). While patients with resectable disease and no evidence of mediastinal lymph node involvement have surgery as the primary treatment, patients with N2 disease usually undergo a multimodality approach in order to maximize treatment outcomes.

Compared with cervical mediastinoscopy, PET-CT has the advantage of being a non-invasive staging method that is becoming increasingly available and has solid data regarding its accuracy. Nevertheless, the pivotal studies were undertaken in areas without endemic cases of tuberculosis and other infectious granulomatous disease [13, 14].

The role of PET-CT in mediastinal staging has been reviewed in a Cochrane meta-analysis [15], which included 18 studies that used 18F-FDG uptake higher than the background activity as qualitative criteria for PET-CT positivity. Sensitivity and specificity estimates were 77.4% (95% CI 65.3 to 86.1) and 90.1% (95% CI 85.3 to 93.5), respectively.

However, some clinicopathological factors have been associated with incorrect PET/CT staging. On multivariate analysis, Al Sarraf [16] showed that rheumatoid arthritis, non-insulin dependent diabetes, history of tuberculosis, presence of atypical adenomatous hyperplasia and pneumonia were independent factors causing inaccurate staging of mediastinal lymph nodes.

An important factor in countries with high burden of granulomatous infectious disease is the reduction of PET-CT reliability in this scenario [17, 18]. According to the World Health Organization (WHO), Brazil ranks as one of the top 20 countries in terms of tuberculosis incidence [19]. Particularly, Porto Alegre, the city where this study has taken place, has an incidence rate of 99,3 cases per 100.000 population [20].

Our report shows that no major impact in sensitivity is seen in an area endemic for tuberculosis and is similar to the literature [15]. On the other hand, specificity is clearly affected, even when higher SUV_max cut off was used. On the per-patient analysis, for SUV_max > 0 we estimated specificity and positive predictive value equal to 0,45 and 0,37, respectively. When a higher cut off was used (SUV_max ≥5), specificity and positive predictive value increased to 0,79 and 0,54; respectively. We also found that for the same SUV cut off (≥ 5), the per-nodal station specificity was slightly higher than per-patient evaluation (0.91 vs 0.79, respectively). This finding is consistent with previous reports showing a decrease in PET-CT specificity when considering only 18F-FDG uptake as qualitative criteria for a positive exam [21–23]. Kim [23] and Lee [21] have reported two cohorts from South Korea, an endemic country for tuberculosis, with specificity of 0,84 and 0,73, respectively. Nonetheless, both studies performed a secondary analysis that only considered positive mediastinal lymph nodes with 18F-FDG uptake without associated calcification or high attenuation. This secondary analysis showed improved specificity of 0,96 and 0,89; respectively.

Additionally, dual time point PET-CT scanning for mediastinal node staging in NSCLC is still controversial. Although some studies have reported that it may be helpful in differentiating malignancy from benign processes, most studies have demonstrated significant overlap of FGD uptake patterns between benign and malignant lesions on delayed time point images [24–26]. We found a higher PET-CT’s positivity for the same SUV cut-offs, although not clinically relevant, which is consistent with previous reports [27].

Our study has some limitations. First, PET-CT has been compared against invasive staging and not the final pathologic report after surgery for all patients. Since surgery was not performed in some of the patients diagnosed with N2 disease, we did not have the pathological specimen after surgery of all patients. Second, the sensitivity found in this report is higher than the reported in the Cochrane meta-nalysis, this could be explained by the number of patients included. Schmidt-Hansen [15] found a significantly higher sensitivity in studies with < 100 participants compared with studies with 100 to 199 participants.

## Conclusions

In conclusion, our findings are in line with the most recent publications and guidelines, which recommend that PET-CT must not be solely used as a tool to mediastinal staging, even in a region with high burden of tuberculosis.

## Abbreviations

18F-FDG PET-CT: Positron emission tomography with 2-deoxy-2-[fluorine-18]fluoro- D-glucose integrated with computed tomography
CNPq: National Council for Scientific and Technological Development
CS: Clinical stage
CT: Computed tomography
HIV: Human immunodeficiency virus
INCA: Brazilian National Institute of Cancer
kV: Kilovolt
LACOG: Latin American Cooperative Oncology Group
mA: Miliampere
mCi: Millicurie
mg: Milligram
ml: Milliliter
NSCLC: Non-small cell lung cancer
OS-EM: Ordered-subset expectation-maximization
ROC: Receiver operator characteristic
STARD: Essential items for reporting diagnostic accuracy studies
SUV: Standardized uptake value
SUV_max: Maximum standardized uptake value
TB: Tuberculosis
WHO: World Health Organization
